# Septic Thrombophlebitis Caused by *Fusobacterium necrophorum* in an Intravenous Drug User

**DOI:** 10.1155/2013/870846

**Published:** 2013-04-16

**Authors:** D. Dimitropoulou, M. Lagadinou, T. Papayiannis, V. Siabi, C. A. Gogos, M. Marangos

**Affiliations:** ^1^Department of Infectious Diseases, University of Patras, 26504 Patras, Greece; ^2^Department of Internal Medicine, University of Patras, 26504 Patras, Greece; ^3^University Hospital of Patras, Rion, 26504 Patras, Greece

## Abstract

Septic thrombophlebitis is characterized by venous thrombosis, inflammation and bacteremia, that can lead to fatal complications such as sepsis, septic emboli and even death. Though most commonly caused by indwelling catheters, it is also related to intravenous drug users (IVDU) especially those who attempt to inject drugs into more proximal and central veins. Lemierre's syndrome, also referred to as post-anginal sepsis or necrobacillosis, is a suppurative thrombophlebitis of the internal jugular vein. Primary infection is associated with oropharyngeal and dental infections and the most common causative organism is *Fusobacterium necrophorum*. We report a case of Lemierre's syndrome in an IVDU, caused by *Fusobacterium necrophorum*, which was inoculated at the site of injection, without a history of sore throat or pharyngitis.

## 1. Introduction

Septic thrombophlebitis is characterized by venous thrombosis, inflammation, and bacteremia, that can lead to fatal complications such as sepsis, septic emboli, and even death [1]. Though most commonly caused by indwelling catheters, it is also related to intravenous drug users (IVDUs), especially those who attempt to inject drugs into more proximal and central veins [2]. The most frequent predominant pathogens of septic thrombophlebitis in IVDUs are *Staphylococcus Aureus* (MSSA or MRSA), followed by *Streptococci* species [1, 3].

Lemierre's syndrome, also referred to as postanginal sepsis or necrobacillosis, is a suppurative thrombophlebitis of the internal jugular vein. Primary infection is associated with oropharyngeal and dental infections, and the most common causative organism is *Fusobacterium necrophorum*, which is part of human microflora of the oropharynx, genitourinary and gastrointestinal tracts.

We report a case of Lemierre's syndrome in an IVDU, caused by* Fusobacterium necrophorum*, which was inoculated at the site of injection, without a history of sore throat or pharyngitis.

## 2. Case Report

**Figure 1:**
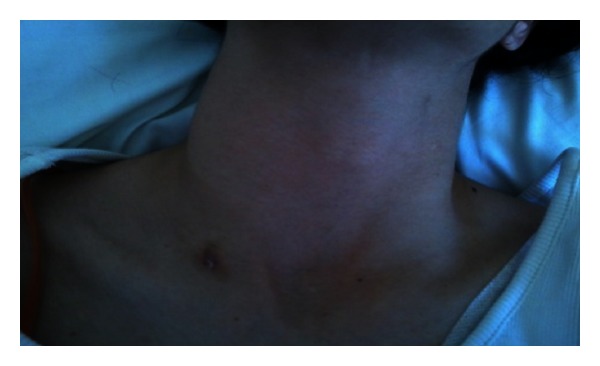
Patient's neck showing tenderness, redness and edema.

A 31-year-old female presented to the emergency department of our hospital, complaining of high-grade fever with chills and shortness of breath while she had been well until two days earlier. She is refered with intravenous heroin use for more than fifteen years. The temperature was 39°C, the pulse rate was 95/min and the respirations were 25/min. Her physical examination revealed tenderness at the right site of the neck, redness and edema with mild regional lymphadenopathy (Figure 1). Examination of the oropharyngeal cavity, tonsils, and lungs was unremarkable. Nuchal rigidity was not noticed. One hour after admission, she became hypotensive requiring vigorous intravenous fluid administration.

The patient mentioned, that during the last 10 months trying to gain a venous access, she frequently used the jugular veins. She denied any needle sharing or using of contaminated syringes. Her usual practice is to lubricate the needle with her saliva and injecting without cleaning the skin.

On routine laboratory testing, the hemoglobulin was 10 g per deciliter, the white cell count was 12200 per cubic millimeter with 90 percent neutrophilia, and elevated C-reactive protein (CRP: 13 mg/dL, normal limits < 0,5 mg/dL). The blood chemical findings were normal. Serological tests were positive for hepatitis C infection.

**Figure 2:**
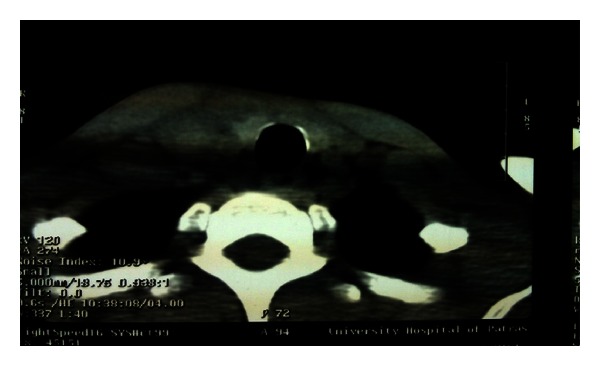
CT showing thrombosis in the right internal jugular vein and soft tissue.

A contrast-enhanced helical computer tomography (CT) of the neck showed extensive thrombus in the right internal jugular vein and enlargement of the regional lymph nodules (Figure 2). CT scan of the thorax and brain was negative. A cardiac ultrasonographic examination was also negative. Based on findings, a diagnosis of Lemierres syndrome was made. Empirical antibiotic treatment with intravenous meropenem, vancomycin (for the possibility of MRSA), and clindamycin was initiated, but clindamycin was discontinued three days later due to gastrointestinal symptoms. The patient became afebrile with clinical improvement. Blood cultures, five days after admission grew *Fusobacterium necrophorum*, and the antibiotic regimen changed to ampicillin-sulbactam plus metronidazole for fourteen days intravenously. At the time of discharge, the patient was in good health and she continued treatment per os for a duration of six weeks. Anticoagulation therapy was initiated with the diagnosis but was discontinued after hospital discharge because of poor compliance.

## 3. Discussion

Bacterial infections are a common complication of intravenous drug use and the reason to being seen in a clinical setting. Skin and soft tissue infections, pneumonia, and endocarditis are most prevalent [3]. Septic thrombophlebitis is a frequent infection of drug users whose peripheral and superficial veins become obliterated, and they have to use more central and proximal veins trying to gain a venous access [2]. 

It is mostly caused by dermal inoculation of pathogens such as *Staphylococcus aureus* and *Streptococcus pyogenes*. Also the substances used to dilute the drug (dextrose, caffeine, lactose, etc.) or to mix with before injecting (water, lemon juice, etc.) may predispose to other pathogens such as *Pseudomonas aeruginosa, Burkholderia cepacia,* and fungi. Other practices, such as diluting the drug or lubricating the needle with saliva, may cause infections by oral flora pathogens.

Our patient used to leak the needle before injecting. Because of this practice, she directly inoculated the microorganism (*Fusobacterium necrophorum*) into her vein causing septicemia.

*Fusobacterium necrophorum* is an anaerobic nonmobile filamentous nonspore forming Gram-negative bacilli which is part of normal oral flora. It is the most common anaerobe in sepsis originating from the oropharynx and it is the predominant pathogen causing Lemierre's syndrome [4–8]. Primary infection of Lemierre's syndrome is associated with pharyngitis, tonsillitis, or dental infection, which is followed by local invasion to the jugular veins causing septic thrombophlebitis [6–8]. To the best of our knowledge, there are no others reports of Lemierre's syndrome from *Fusobacterium necrophorum* originated after direct inoculation of the pathogen into the vein.

In our case, diagnosis of septic thrombophlebitis was suspected because of the history of IV drug use, local signs of thrombosis and inflammation, positive blood cultures together with radiological findings consistent with venous obstruction. Imaging studies alone (contrast enhanced computer tomography or Doppler ultrasonography) can be helpful for the diagnosis but cannot distinguish between septic or non septic thrombophlebitis [9].

The choice of initial antibiotics should be based on clinical status, predisposing factors and the most common pathogens [10]. Therefore, as the patient was septic, our empiric therapy included meropenem, clindamycin, and vancomycin. Based on the results of blood cultures, we simplified the regimen to ampicillin-sulbactam plus metronidazole for a total duration of 6 weeks which is the recommended duration for Lemierre's syndrome.

Pulmonary emboli and endocarditis must always be suspected in cases with clinical deterioration besides the administration of appropriate antibiotic regimens. The benefit of anticoagulation therapy for the management of Lemierre's syndrome is uncertain.

In conclusion, septic thrombophlebitis is a frequent infection in IVDU with high mortality rates. Clinicians must be very careful for early recognition of clinical signs. Case history and daily practices of the patient must always be considered for the choice of initial antibiotic therapy.

## Abbreviations

IVDU: Intravenous drug user
MSSA: Methicillin sensitive *Staphylococcus aureus *
MRSA: Methicillin-resistant *Staphylococcus aureus *
WBC: White blood cell
CRP: C-reactive protein
CT: Computer tomography.
